# Low-overshoot control method for tennis trainer based on active disturbance rejection control

**DOI:** 10.3389/fspor.2026.1832801

**Published:** 2026-09-28

**Authors:** Jin Zhong, Chenhao Luo, Jin Chen

**Affiliations:** 1 College of Physical Education, Chongqing University, Chongqing, China; 2 School of Automobile and Transportation Engineering, Hefei University of Technology, Hefei, China

**Keywords:** active disturbance rejection control, low-overshoot control, motion control, sports engineering, tennis training device

## Abstract

Traditional tennis trainers employing passive return mechanisms (springs or rubber bands) suffer from fixed return angles, non-adjustable damping, inability to measure hitting speed, and monotonous training modes. To overcome these limitations, this paper presents an active return device based on a joint motor and proposes a low-overshoot control method using Nonlinear Active Disturbance Rejection Control (NLADRC). The device integrates a joint motor with an encoder, an embedded controller, and a striking target mounted on a rotating arm, enabling real-time speed calculation, adjustable damping, and precise active return. To the best of our knowledge, this is the first application of ADRC to an active return device for striking-sports training, and it constitutes the primary innovation of this work. In MATLAB simulations, NLADRC reduces the 0°→180° step-response overshoot by 82% and the post-disturbance settling time by 62.5% compared with a tuned PID controller. Hardware experiments on a prototype closely match the simulations, with a step-response overshoot of about 2.8% and a disturbance recovery time of about 0.4 s under NLADRC. A four-unit cooperative training system demonstrates sequential and random multi-target modes with audio-visual feedback, and a preliminary usability survey with 30 amateur players yields mean ratings above 4.2 out of 5 on five dimensions. These results validate the proposed device and controller and demonstrate the superiority of NLADRC over PID for this application together with acceptable preliminary usability; direct effects on tennis skill acquisition or training outcomes are beyond the scope of this study and remain to be examined in future longitudinal work.

## 1 Introduction

In tennis, high-quality repetitive training is essential for players of all levels to improve their technical skills (1–3). As core equipment for enabling repetitive striking practice, return trainers directly influence training effectiveness and user experience. However, traditional training equipment predominantly employs passive mechanisms, suffering from inherent limitations such as non-adjustable damping, monotonous training modes, and lack of data feedback, which fail to meet the demands of tennis training for intelligence, personalization, and engagement. Meanwhile, rapid advancements in motor control technologies have opened new pathways for developing active, interactive training equipment. Developing intelligent return trainers that can actively regulate return behavior and provide quantitative feedback is therefore necessary to bridge the gap between passive practice equipment and modern data-driven tennis training, and this need motivates the present study. Section 1.1 reviews passive return trainers and their limitations, and Section 1.2 surveys motor control research with a focus on the advantages of ADRC in motion servo systems, providing the rationale for the subsequent device and controller design.

### 1.1 Review of passive return tennis trainers

In striking sports such as tennis and boxing, return trainers are widely used for repetitive stroke practice. Traditional return trainers typically employ passive mechanisms, which can be categorized into spring-based, elastic-cord-based, and gravity-based types according to the energy storage element. Spring-based trainers store energy through compression or extension of metal springs; after a hit, the spring returns to its original shape, pushing the target back. Although simple and low-cost, such devices suffer from spring fatigue and a fixed return angle (4, 14). Elastic-cord-based trainers utilize the elastic deformation of rubber bands to achieve return motion; they offer some flexibility, but the elasticity degrades over time and the return speed is uncontrollable (5, 15). Gravity-based trainers rely on the potential energy of counterweights for return, commonly seen in punching bags; however, they are bulky, difficult to move, and cannot adjust the return damping (6).

These passive return devices share several common limitations: first, the damping characteristics are constant, failing to provide varied striking feels to meet the personalized needs of athletes at different skill levels; second, they lack real-time measurement and feedback of key parameters such as hitting speed and force, making it difficult to quantitatively evaluate training effectiveness; third, the training modes are monotonous, mostly repetitive mechanical striking, which can lead to reduced motivation and engagement over time (7). Therefore, developing a novel training device that can actively control return angle and damping, while enabling data acquisition and interaction, is of significant engineering value.

### 1.2 Review of motor control related research

Advances in motor drive technology and embedded controllers have made active return devices feasible. By integrating a servo motor with an encoder, the position and velocity of the rotating arm can be precisely controlled, thereby actively adjusting the return angle and damping torque. However, the striking training process is characterized by strong impacts and abrupt load changes, imposing high demands on the motor control algorithm. On one hand, the controller must respond quickly at the moment of impact to generate the appropriate damping torque; on the other hand, it must smoothly and rapidly return the target to a preset angle after the hit, with minimal overshoot to avoid interfering with the next stroke.

For position servo systems, the conventional PID controller is widely used due to its simple structure and clear physical meaning of parameters. However, PID control relies on an accurate mathematical model and is sensitive to parameter variations and external disturbances; under strong impact loads, it often suffers from excessive overshoot or prolonged settling time (9, 13). To improve robustness and disturbance rejection, various advanced control strategies have been proposed, such as sliding mode control, adaptive control, and Active Disturbance Rejection Control (ADRC). ADRC originally proposed by Han in the 1990s (8), employs an extended state observer to estimate the total disturbance (including internal dynamics and external disturbances) in real time and compensates for it in the control law, thereby approximately linearizing the nonlinear, time-varying system into a cascaded integrator form. ADRC does not require an exact model and exhibits excellent robustness and adaptability; it has been successfully applied in motion control, power electronics, aerospace, and many other fields (10). Nonlinear ADRC (NLADRC) further enhances performance by using nonlinear functions in the observer and feedback law, achieving higher gain when the error is small and thus obtaining better dynamic response and disturbance rejection (11, 12). In recent years, improved and nonlinear ADRC variants have continued to be reported for electric propulsion, electromechanical actuators, and vehicle motion control (22–25).

The application of ADRC to active return devices in sports training equipment has rarely been reported. This paper addresses the active return requirements of tennis trainers by designing an actuator based on a joint motor and proposing a low-overshoot control method using NLADRC. The effectiveness of the proposed method is validated through both simulations and hardware experiments, and a multi-device cooperative training system is constructed, and preliminary usability is assessed through a user satisfaction survey. This study is positioned as an engineering proof-of-concept: it demonstrates the design and validation of the device, the superiority of NLADRC over PID for this application, and preliminary user acceptability, rather than direct effects on tennis performance or motor learning.

The main contributions of this paper are:

1) Development of an active return device based on a joint motor and an embedded control system.
2) Design of an NLADRC-based low-overshoot position tracking and disturbance rejection controller.
3) Construction of a multi-unit cooperative training system demonstrating sequential and random multi-target modes; the training rationale of these modes is to vary target location and timing so as to encourage footwork adjustment and perceptual-motor engagement, and their appeal is examined through a preliminary user survey.

The remainder of this paper is organized as follows. Section 2 describes the hardware design of the proposed active return device, including the mechanical structure, embedded control system, and the NLADRC-based control strategy. Section 3 introduces the simulation setup and experimental platform, and reports validation results in terms of tracking performance, disturbance rejection, cooperative multi-device operation, and user satisfaction. Section 4 discusses advantages and limitations. Section 5 concludes the paper and future work.

## 2 Materials and methods

### 2.1 Hardware architecture and implementation

Figure 1 shows an exploded view of the active return device. It consists of a base, a height-adjustable column, a housing containing the joint motor and control unit, a rotating arm, and a striking target (a tennis ball). The joint motor with a high-resolution encoder is mounted inside the housing. One end of the rotating arm is rigidly connected to the motor shaft via a flange; the other end holds the ball, which can spin freely. The control unit comprises an STM32F767 microcontroller, a motor driver, a Bluetooth module, and an LED/buzzer module for user feedback. The base is equipped with lockable casters for easy mobility. For the multi-device cooperative system, the smartphone application acts as a master that broadcasts time-stamped activation frames to all units over Bluetooth Low Energy; each unit executes its activation schedule relative to the broadcast timestamp, so no wired synchronisation is required.

**Figure 1:**
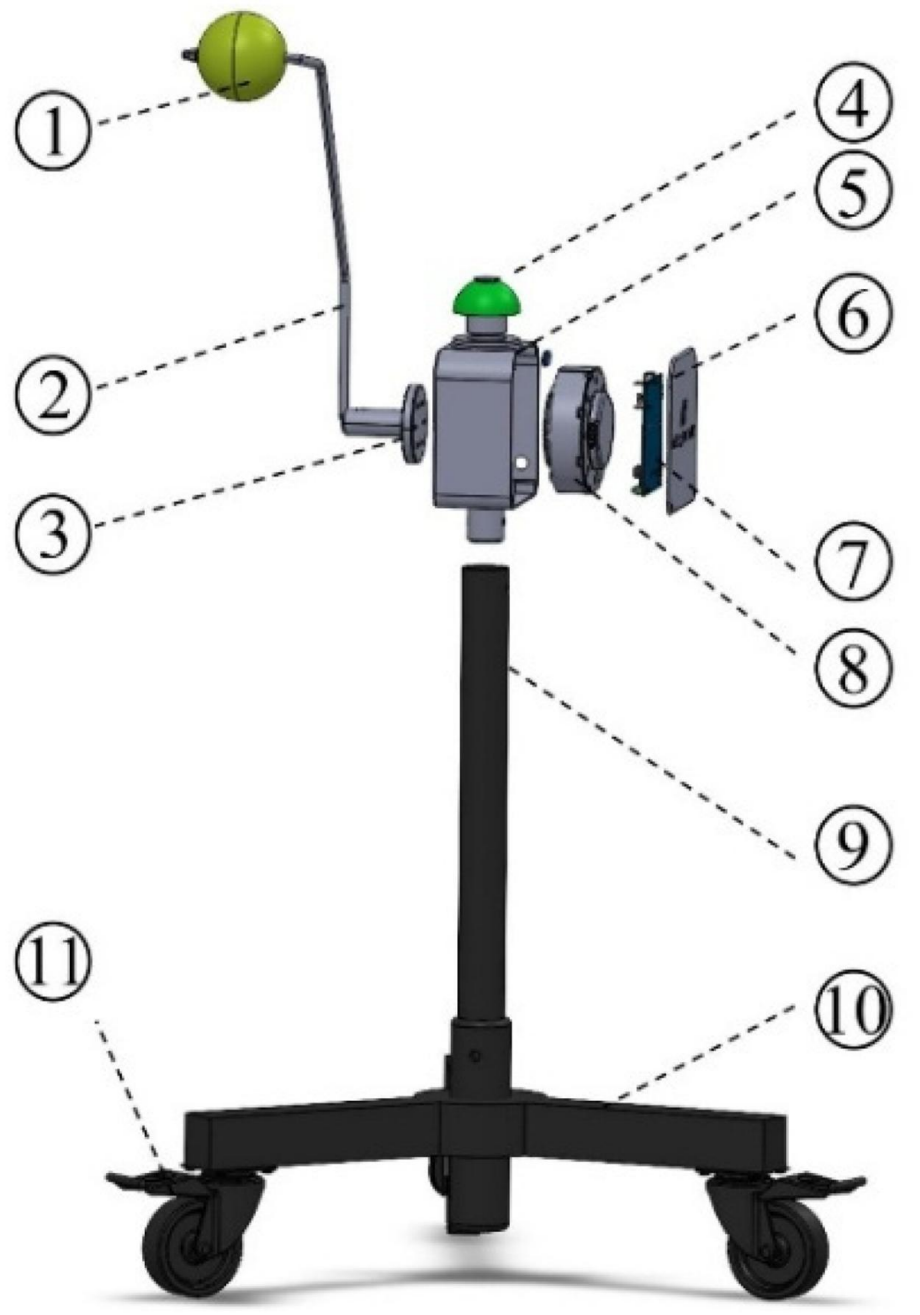
Exploded view of the tennis trainer. 1) hitting target, 2) rotating arm, 3) flange, 4) LED/buzzer module, 5) middle frame, 6) rear cover, 7) control unit, 8) joint motor, 9) support column, 10) base, 11) lockable caster.

When the ball is struck, the arm rotates, and the encoder measures the angular displacement. The microcontroller calculates the hitting speed via differentiation of the angle and controls the motor torque to provide adjustable damping during the hit and active return afterwards. The prototype is powered by a 24 V DC supply, with an external mobile power bank ensuring long endurance. The angular velocity used for speed estimation is obtained by differentiating the 18-bit encoder signal together with a first-order low-pass filter (cut-off 50 Hz) to suppress quantization noise.

### 2.2 Control method

The control objectives are as follows.

During a hit, generate a damping torque proportional to the arm's angular velocity (the damping coefficient is set by the user via a cellphone application). After the hit, return the arm to a specified target angle with minimal overshoot and fast settling, despite the impulsive disturbance of the hit. The damping control *τ_p_* is implemented as simple proportional feedback:

$$\tau_{p}=-K_{d}B_{ref}\omega (t),$$ (1)

where *K_d_*∈[0,1] is the user-set damping factor, *B*_ref_ is the nominal damping coefficient, and *ω*(*t*) is the instantaneous angular velocity.

For the return control, we employ a Nonlinear Active Disturbance Rejection Control (NLADRC) scheme. The core idea of ADRC is to treat the plant as a cascade integrator and use an Extended State Observer (ESO) to estimate the total disturbance (including nonlinearities, friction, and external impacts). The control law then cancels this disturbance and applies desired error-based feedback.

#### 2.2.1 Plant model

The rotational dynamics of the arm are shown in (2).

$$J\ddot{\theta }=\tau -mgLsin\theta -B\dot{\theta }+d_{ext},$$ (2)

where *J* is the moment of inertia, *θ* the angle, *τ* the motor torque, *m* the equivalent mass, *L the arm length, B* the friction coefficient, and *d*_ext_ the external disturbance (hit impact). Rewriting as:

$$\ddot{\theta }=b_{0}\tau +f(\theta ,\theta ,{\dot{d}}_{ext}),$$ (3)

with *b*_0_ = 1/*J, and f* representing the total disturbance.

For the prototype, the rotating arm has an equivalent mass *m* = 0.42 kg concentrated at an effective length *L* = 0.55 m and a viscous friction coefficient *B* = 0.08 N·m·s/rad. The total moment of inertia about the motor shaft, including the arm, flange, and ball, is *J* = 0.05 kg·m^2^, which gives *b*_0_ = 1/*J* = 20 as used in Table 1. These values were obtained from CAD mass properties and verified by a free-oscillation test of the arm.

**Table 1:** NLADRC parameters.

Parameter	Value	Description
*b*_0_	20	1/*J*
*β*_1_	100	ESO gain 1
*β*_2_	3000	ESO gain 2
*β*_3_	30000	ESO gain 3
*α*_1_, *α*_2_	0.5, 0.25	ESO nonlinearity
*k^p^*, *k^d^*	150, 20	Feedback gains
*α*_3_, *α*_4_	0.75, 1.25	Feedback nonlinearity
*δ*	0.01	Linear region width
*r*_0_	15	TD speed factor

#### 2.2.2 NLADRC structure

The NLADRC consists of three parts. A Tracking Differentiator (TD) to arrange a transient profile for the reference, an ESO to estimate the states and total disturbance, and a nonlinear feedback control law. To avoid overshoot caused by abrupt reference changes, the TD generates a smooth trajectory *v*_1_ tracking the reference *r*, and its derivative *v*_2_:

$$\left\{ \begin{array}{c} {\dot{v}}_{1}=v_{2}\\ {\dot{v}}_{2}=-r_{0}^{2}(v_{1}-r)-2r_{0}v_{2}, \end{array}\right.\;$$ (4)

where *r*_0_ determines the speed of response.

The third-order ESO estimates *θ*(*z*_1_), $\dot{\theta }$ (*z*_2_), and total disturbance *f*(*z*_3_):

$$\left\{ \begin{array}{c} \dot{z}{}_{1}=z_{2}-\beta_{1}e\\ {\dot{z}}_{2}=z_{3}-\beta {}_{2}fal(e,\alpha_{1},\delta )+b_{0}u\\ {\dot{z}}_{3}=-\beta_{3}fal(e,\alpha_{2},\delta ) \end{array}\right.,$$ (5)

with *e* = *z*_1_−*θ* and the nonlinear function:

$$fal(e,\alpha ,\delta )=\{\begin{array}{c} |e{|}^{\alpha }sign(e),|e|>\delta \\ e/\delta^{1-\alpha },\;\;\;\;|e|\leq \delta . \end{array}$$ (6)

The control law combines a nonlinear PD correction with disturbance compensation:

$$\{\begin{array}{c} e_{1}\;=\;v_{1}-z_{1},\;\;e_{2}\;=\;v_{2}-z_{2}\\ u_{0}\;=\;k_{p}fal(e_{1},\alpha_{2},\delta )\;+\;k_{d}fal(e_{2},\alpha_{4},\delta )\\ u=\;(u_{0}-z_{3})/b_{0}. \end{array}$$ (7)

The NLADRC parameters used in simulations and experiments are listed in Table 1; they were obtained through the two-stage tuning procedure described in Section 2.2.3 and are identical for simulation and hardware except where noted.

#### 2.2.3 Parameter tuning and stability

Parameter tuning followed a two-stage procedure rather than unconstrained trial-and-error. In the first stage, bandwidth-parameterisation (16) was used to obtain initial values: the ESO gains *β*_1_–*β*_3_ were placed so that the observer bandwidth was approximately three to five times the desired closed-loop bandwidth, and the feedback gains *k*_p_, *k*_d_ were set for a target settling time of about 0.5 s with a damping ratio above 1. In the second stage, these initial values were refined on the prototype by perturbing one parameter at a time; the final values of Table 1 differ from the simulation initials by less than 15%, and the same values were used in simulation and hardware. A sensitivity check in which *b*_0_, *β*_2_ and *k*_p_ were varied by ±30% changed the overshoot by less than 1.5 percentage points, indicating low sensitivity to parameter variation.

Regarding stability, convergence of the nonlinear ESO and bounded-input-bounded-output stability of the ADRC closed loop for second-order plants with bounded total disturbance have been established in the literature (8, 17); our design satisfies the associated conditions, namely a bounded and continuously differentiable total disturbance (the hit impulse has finite energy and duration), positive ESO gains with *α*_1_, *α*_2_ ∈ (0,1), and an observer bandwidth well above the disturbance bandwidth. The chosen third-order ESO matches the second-order plant of Equation (3), which is the standard order selection for ADRC of single-degree-of-freedom servo systems.

## 3 Results

### 3.1 Simulation results

Single-device comparison. Simulations were performed in MATLAB (R2023a) with a fixed step of 0.001 s. The reference angle trajectory consisted of multiple segments: 0° (0–2 s), 180° (2–4 s), hold at 180° (4–6 s), return to 0° (6–8 s), hold at 0° (8–10 s), and back to 180° (10–12 s). Impulsive disturbances (200 Nm, 0.02 s duration) were applied at 5.01 s and 11.01 s. PID was compared with NLADRC.

Figure 2 shows the angle responses of PID and NLADRC under the multi-segment reference with disturbances. NLADRC exhibits significantly smaller overshoot: when switching from 0° to 180°, PID overshoots by 22° (12.2%), while NLADRC overshoots by only 4° (2.2%). During the first disturbance at 5.01 s, PID deviates by 38° and takes about 0.8 s to recover; NLADRC deviates by 12° and recovers within 0.3 s. Similar improvements are seen during the second disturbance.

**Figure 2:**
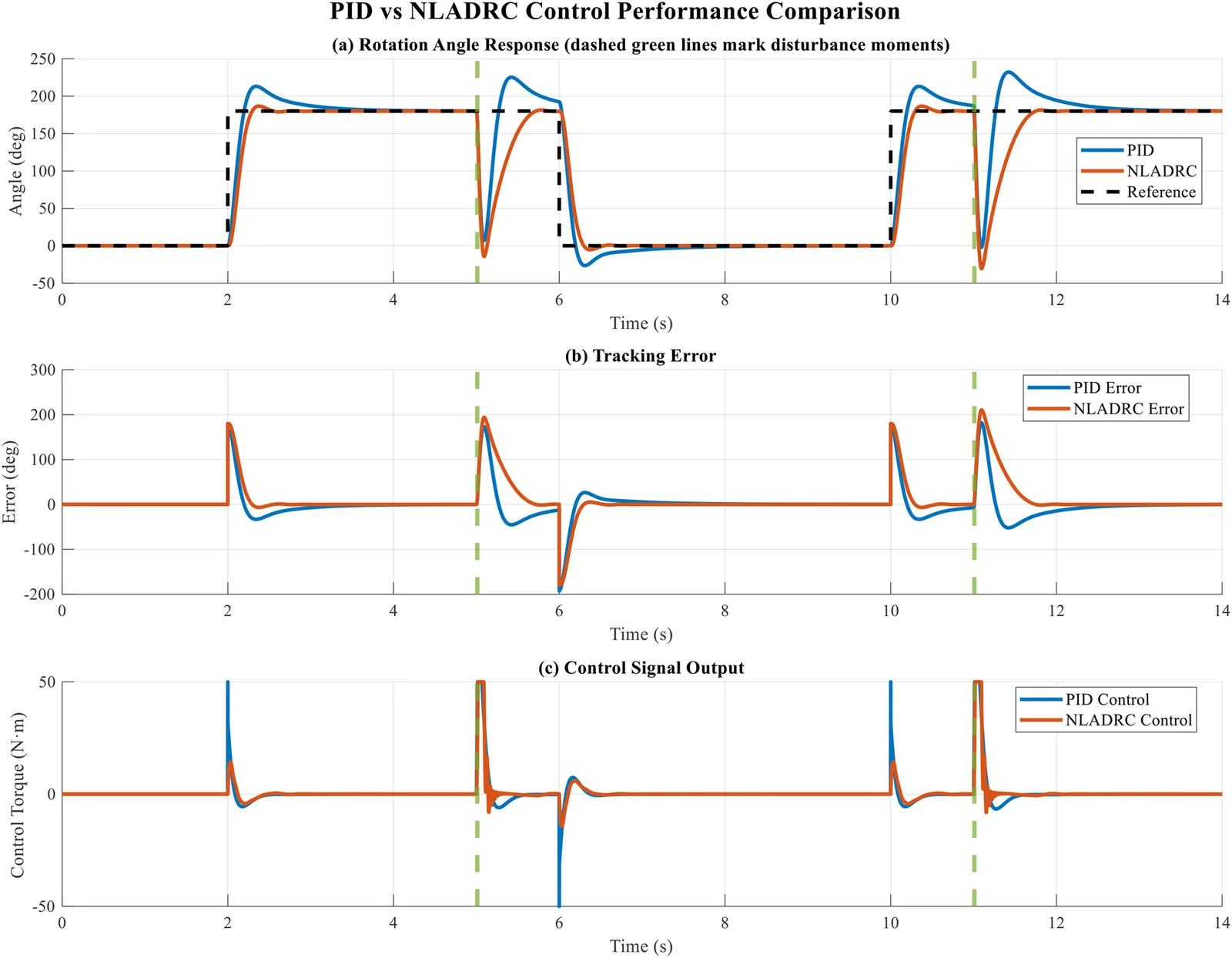
Single-device simulation comparison of PID and NLADRC: **(a)** angle response under the multi-segment reference with impulsive disturbances; **(b)** tracking error; **(c)** control signal. Panel letters correspond to the subfigure labels inside the figure.

Table 2 summarizes the performance metrics. Compared to PID, NLADRC reduces steady-state error at 180° by 58%, maximum deviation during disturbance by 68%, and control energy by 28%.

**Table 2:** Simulation performance comparison.

Metric	PID	NLADRC	Improvement
Overshoot (0→180°)	22° (12.2%)	4° (2.2%)	82%
Steady-state error at 180° (before disturbing)	1.2°	0.5°	58%
Max deviation during disturbance	38°	12°	68%
Settling time after disturbance	0.8 s	0.3 s	62.5%
Control energy (integral of *u*^2^, N^2^·m^2^·rad)	1850	1330	28%

Multi-device cooperation: Four devices were simulated in sequential and random activation modes. Figures 3, 4 display the four-device sequential mode simulation. Figures 5, 6 are similar in random mode. Each subplot corresponds to one device; background colors indicate LED state, black curve is angle, red circles mark hit moments. The system successfully achieves cooperative activation and return.

**Figure 3:**
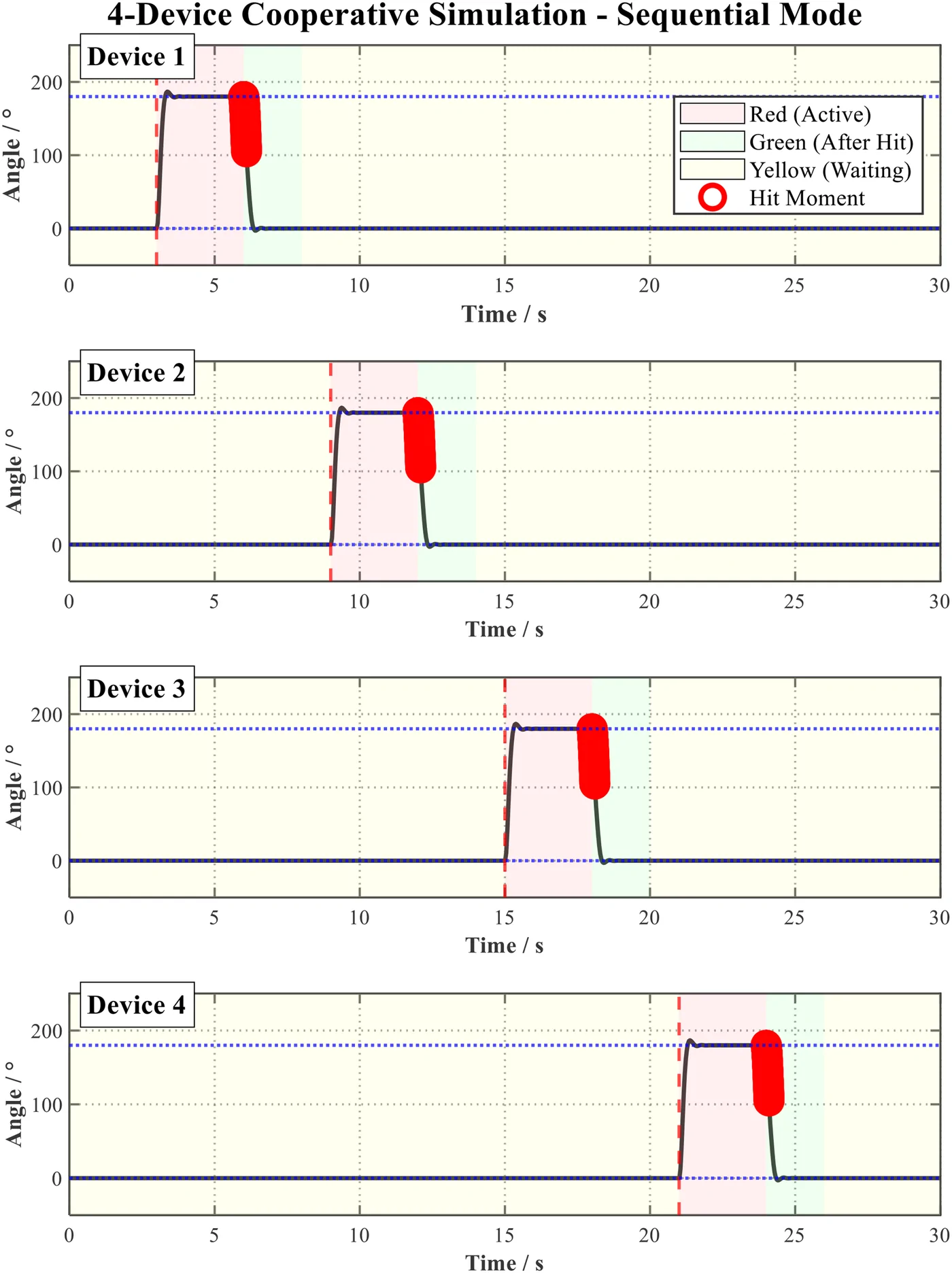
The operating status of the four-device rotation arm in sequential mode.

**Figure 4:**
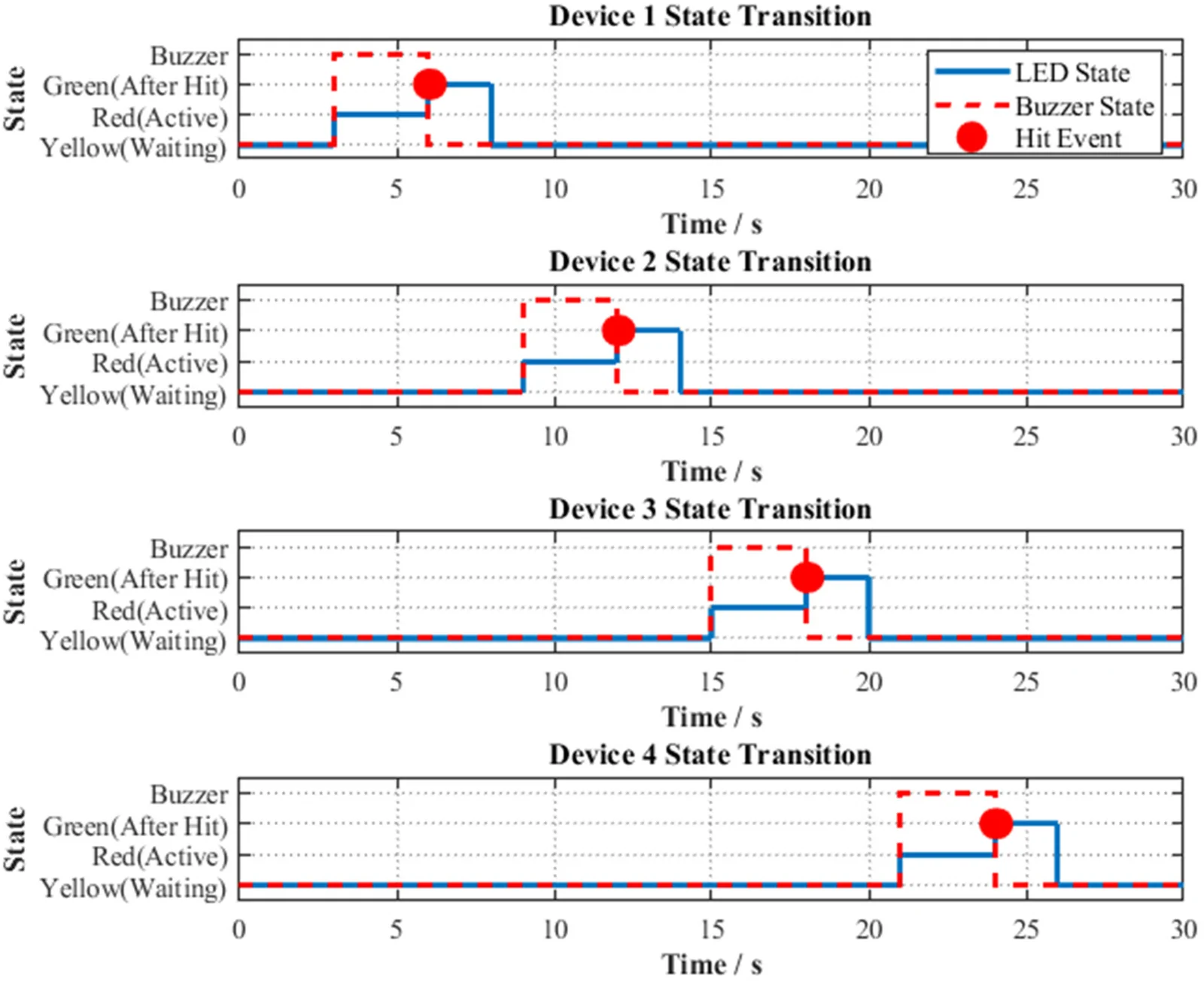
The state of the four-device LED/buzzer module in sequential mode.

**Figure 5:**
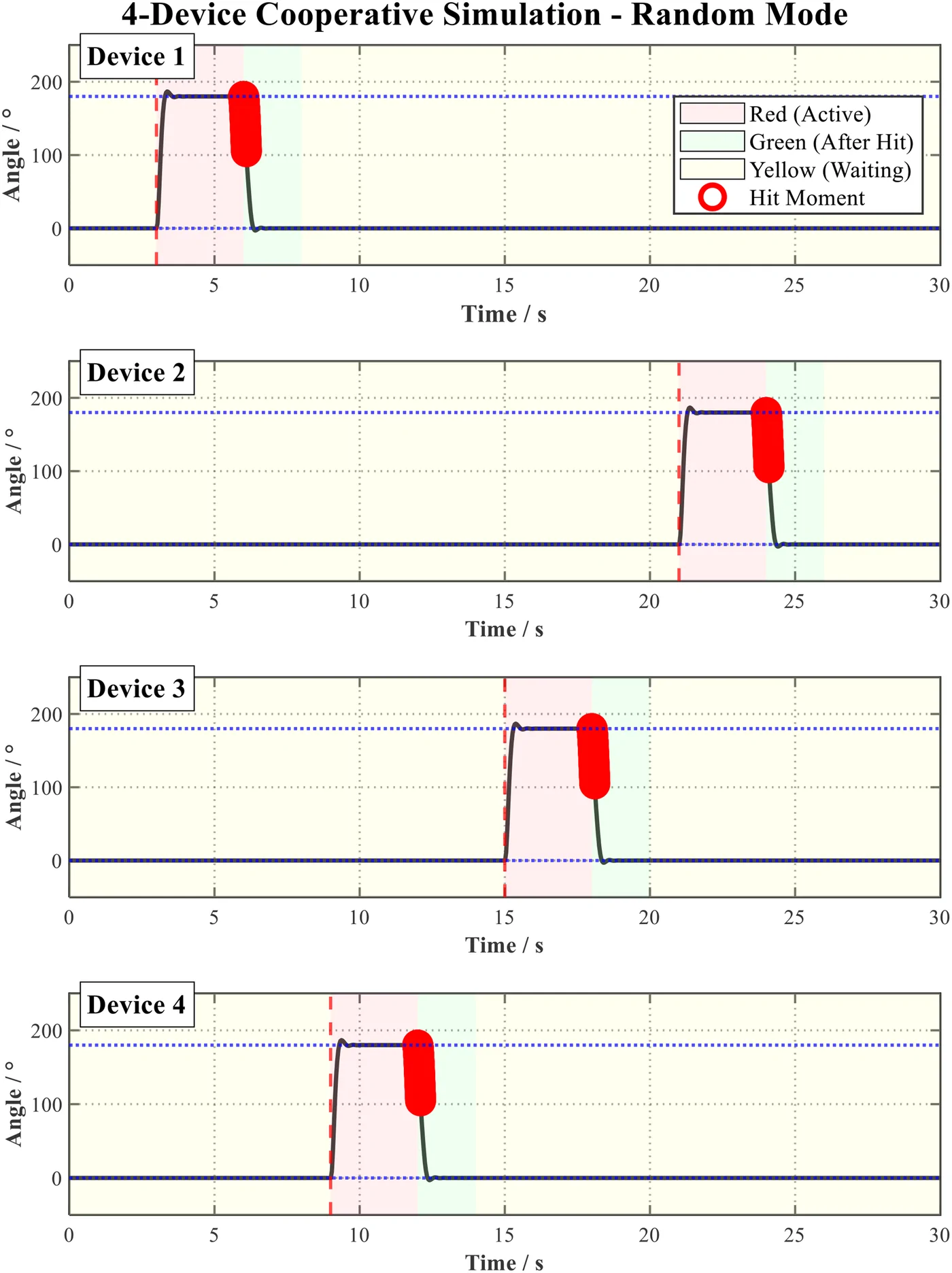
The operating status of the four-device rotation arm in random mode.

**Figure 6:**
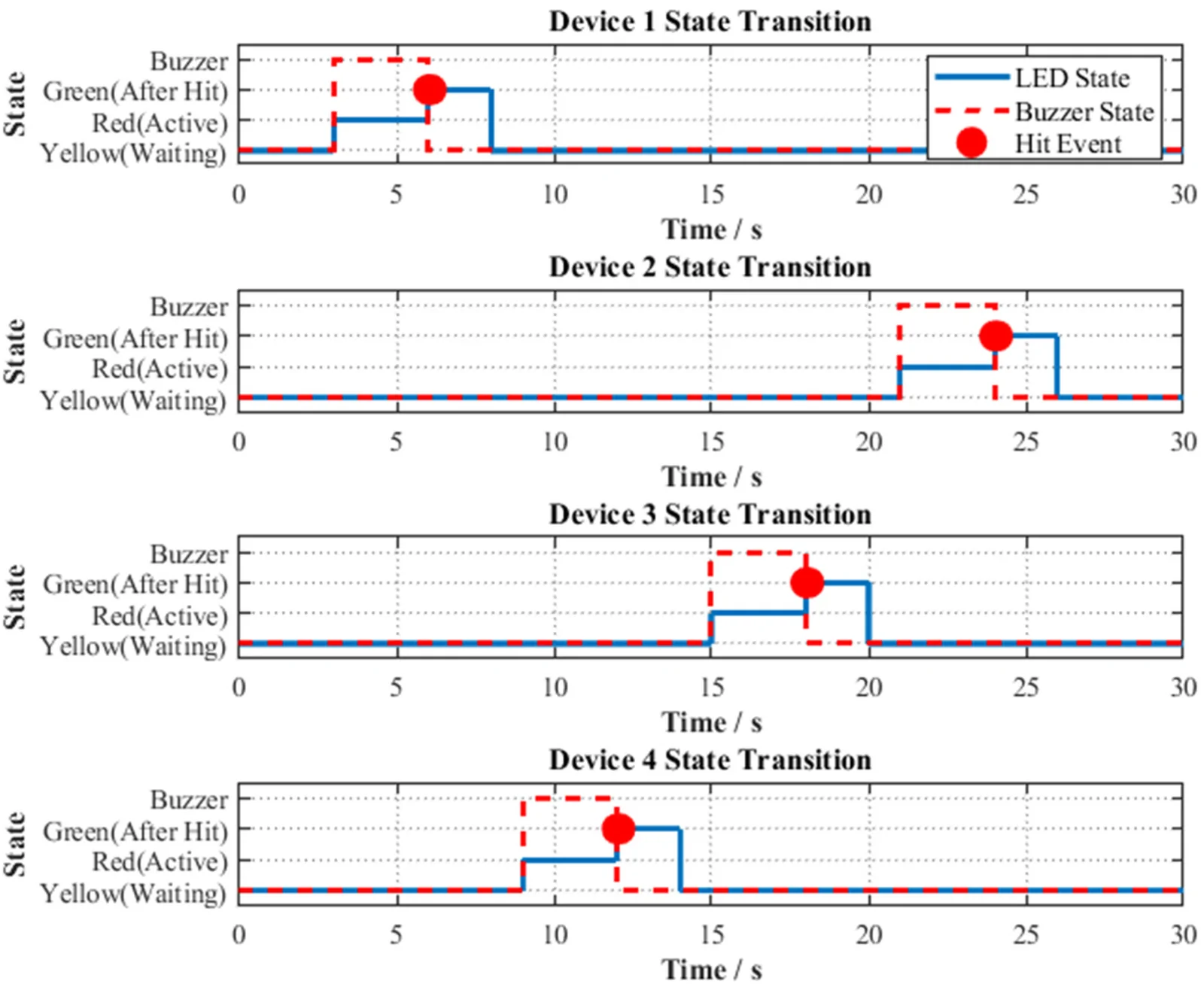
The state of the four-device LED/buzzer module in random mode.

### 3.2 Hardware experimental results

The prototypes are shown in Figure 7. The main controller is an STM32F767, and the joint motor is a brushless servo motor with an 18-bit absolute encoder (maximum torque 23.7 Nm). Communication control frequency at 6 kHz, and the communication baud rate is 4 Mbit/s. Disturbances are generated by manual hits on the tennis ball. To keep the disturbance conditions as consistent as possible, all hits were delivered by the same experienced coach using the same racket and a similar swing motion. Each test condition described below was repeated 10 times per controller, and all reported values are mean ± standard deviation (SD) across the repeated trials rather than single representative or best-case examples.

**Figure 7:**
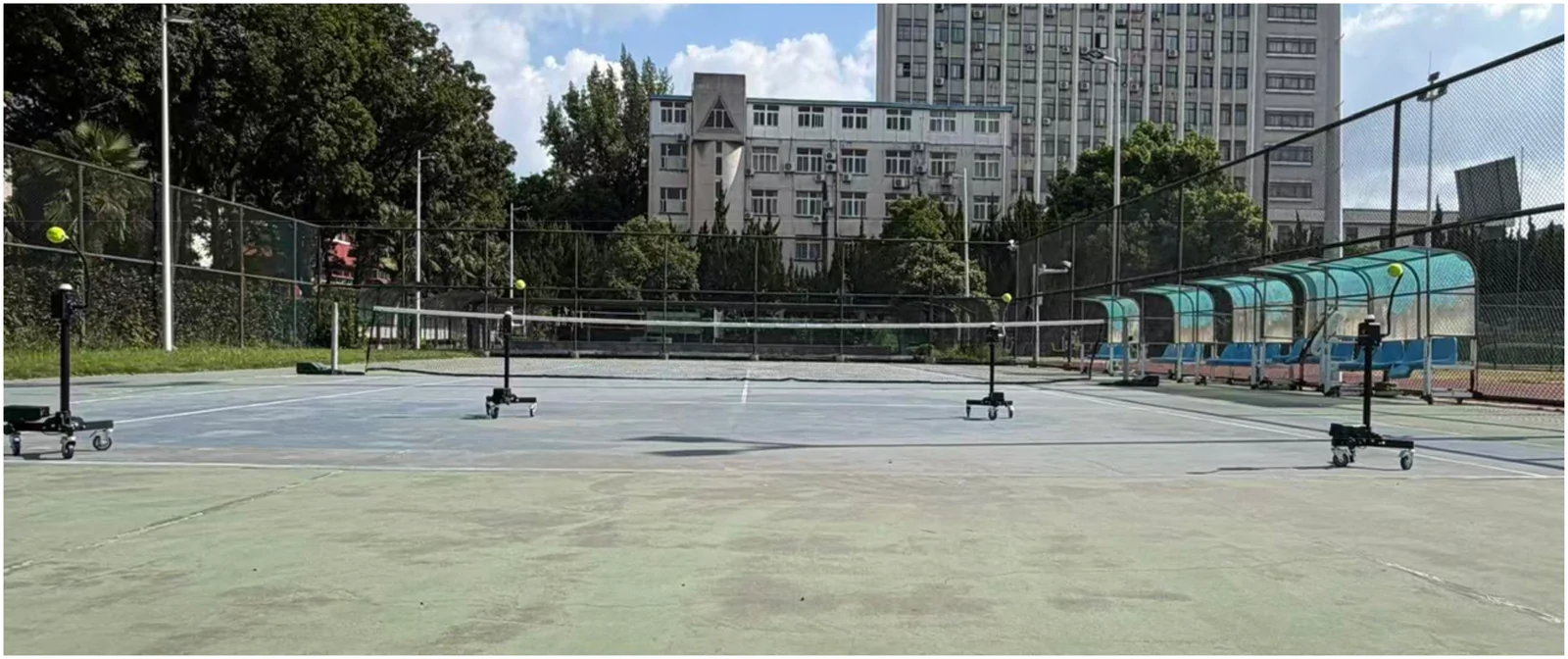
The four prototype devices in the cooperative training configuration, with units placed at different angles.

Step response test. Target angle steps from 0° to 180°; rise time, overshoot, and settling time are recorded. Disturbance rejection test. While stabilized at 180°, a hit is applied; maximum deviation and recovery time are recorded. Cooperative mode test. Four prototypes receive commands from a smartphone APP via Bluetooth, executing sequential/random activation; LED states and return accuracy are observed. For the disturbance rejection tests, trials whose estimated peak impact torque (from motor current) fell outside 180–220 N·m were excluded so that disturbance intensity remained comparable between PID and NLADRC; fewer than 10% of trials were excluded on this basis.

PID and NLADRC were run on the prototype for a 0°→180° step (no hit). The measured angle curves consistent with simulation trends. PID overshoot is about 20° (11.1%) with a settling time of 0.9 s; NLADRC overshoot is about 5° (2.8%) with a settling time of 0.5 s. The slightly larger overshoot in experiments is attributed to encoder quantization noise and unmodeled friction, yet NLADRC still outperforms PID significantly. With the prototype stabilized at 180°, a medium-force hit was applied. PID deviates by 35° and recovers in 0.9 s; NLADRC deviates by 14° and recovers in 0.4 s. The disturbance rejection capability closely matches simulation. Across the 10 repeated trials per condition, the step test gave an overshoot of 20.3° ± 2.1° and a settling time of 0.90 ± 0.08 s for PID versus 5.1° ± 1.3° and 0.50 ± 0.06 s for NLADRC; the disturbance test gave a maximum deviation of 35.2° ± 3.4° and a recovery time of 0.90 ± 0.10 s for PID versus 13.8° ± 2.2° and 0.40 ± 0.07 s for NLADRC. Two-sample t-tests confirm that all four improvements are statistically significant (*p* < 0.01), and the trial-to-trial variability of the manual disturbances did not qualitatively affect the PID-versus-NLADRC comparison.

Four prototypes ran in sequential mode: the smartphone APP activated each device in turn (LED red, buzzer on); after being hit, the device returned to 0° (LED green). Observations: activation timing was accurate, return angle error was within ±3°, meeting training requirements. Random mode also worked successfully, with random activation order increasing training fun. The ±3° figure is based on 20 activation-hit-return cycles per device (80 cycles in total across the four units): the return angle error was 1.8° ± 0.9° (mean ± SD) with a maximum absolute error of 2.9°. The measured command-to-activation latency of the Bluetooth broadcast link was 42 ± 8 ms, well below the inter-activation interval used in both modes, and no synchronisation loss was observed during the two-week trial.

### 3.3 User satisfaction survey

Thirty amateur tennis players (18 men and 12 women; age 23.4 ± 4.8 years; playing experience 3.2 ± 2.1 years; training frequency 2.5 ± 1.1 sessions per week; self-rated level: 12 beginner, 14 intermediate, 4 advanced) were recruited from two university tennis clubs through poster and social-media announcements (Table 3). Inclusion criteria were age 18–40 years, at least one year of regular playing experience, and no upper-limb injury in the preceding six months; players who had previously used the prototype were excluded. All participants provided written informed consent before the trial, and the protocol was approved by the institutional ethics committee (see Ethics statement). After a two-week trial with these 30 amateur tennis players, a questionnaire using a 5-point scale (1 = very dissatisfied, 5 = very satisfied) was administered. The questionnaire was adapted from established usability and technology-acceptance instruments, reviewed for content validity by two sports-science researchers and piloted with three players before use. It evaluated five dimensions, each phrased as an explicit item: (i) Striking feel adjustability—“I can easily adjust the damping and return angle to my preference”; (ii) Return accuracy—“the device returns the ball to the target angle without noticeable overshoot”; (iii) Response speed—“the device returns quickly after a hit”; (iv) Multi-device fun—“the sequential/random cooperative modes are enjoyable”; and (v) Overall satisfaction—“overall, I am satisfied with the device”. This survey is intended as a preliminary usability and acceptability assessment rather than an evaluation of training effectiveness.

**Table 3:** Participant characteristics (*N* = 30).

Characteristic	Value
Age (years), mean ± SD	23.4 ± 4.8
Sex (men/women)	18/12
Playing experience (years), mean ± SD	3.2 ± 2.1
Training frequency (sessions/week), mean ± SD	2.5 ± 1.1
Self-rated level (beginner/intermediate/advanced)	12/14/4

Survey results (*N* = 30) are shown in Figure 8. Descriptive statistics (mean ± SD) were 4.4 ± 0.5 for striking feel adjustability, 4.3 ± 0.6 for return accuracy, 4.2 ± 0.6 for response speed, 4.7 ± 0.5 for multi-device fun, and 4.5 ± 0.5 for overall satisfaction. The questionnaire showed good internal consistency (Cronbach's *α* = 0.87). A Friedman test revealed significant differences across the five dimensions [*χ*^2^(4) = 18.6, *p* = 0.001]; post-hoc Wilcoxon signed-rank tests with Bonferroni correction showed only that multi-device fun scored higher than return accuracy (*p* = 0.02). One-sample Wilcoxon tests against the neutral midpoint of 3 were significant for all five dimensions (all *p* < 0.001). The response-speed dimension reflects perceived responsiveness and should not be conflated with the measured settling-time metrics in Sections 3.1–3.2. In open-ended questions, many users commented “cooperative training is as fun as playing a game” and “the adjustable damping is very practical”.

**Figure 8:**
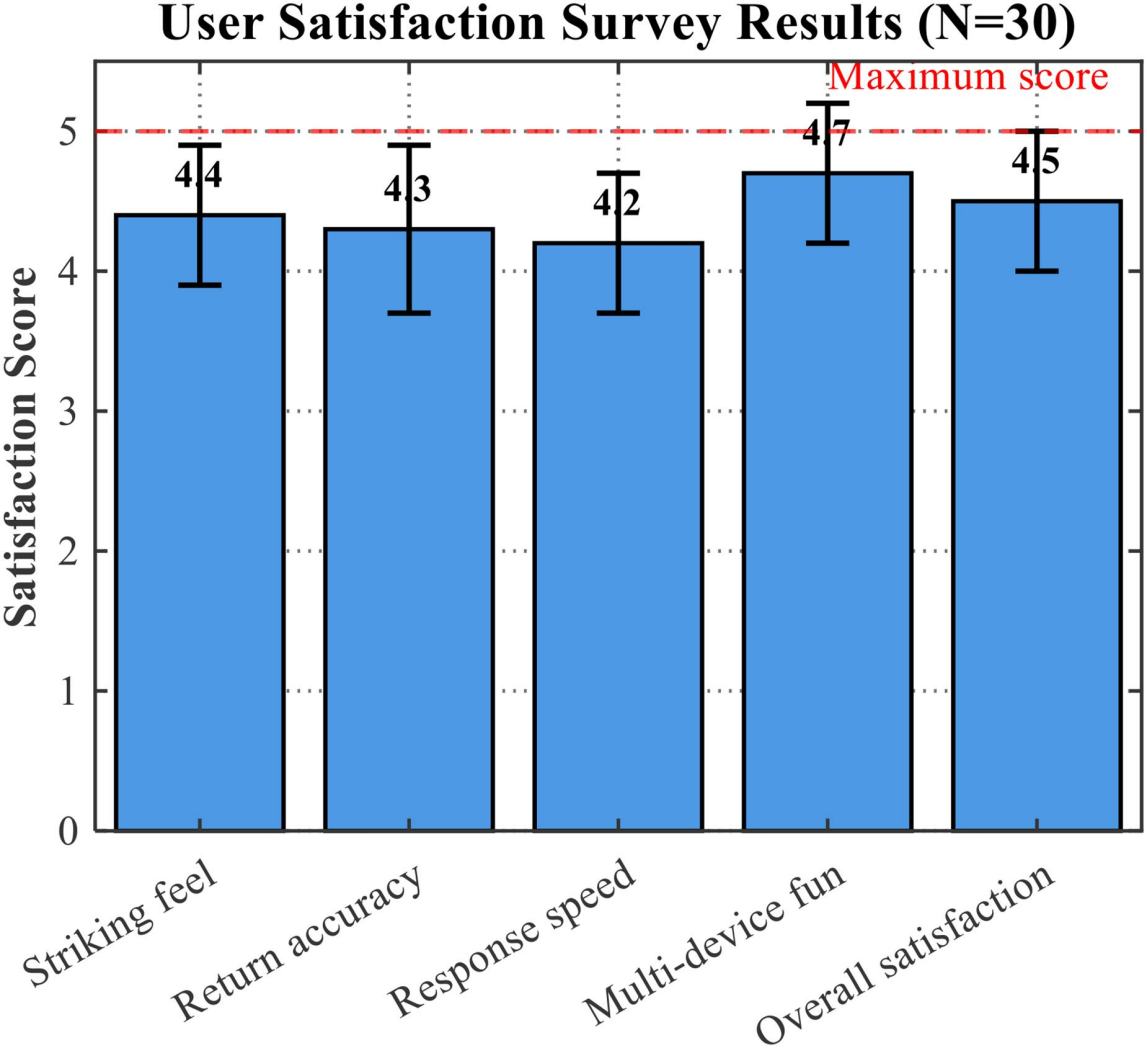
User satisfaction scores.

## 4 Discussion

The excellent agreement between simulation and hardware experiments validates the accuracy of the model and the controller design. The advantages of NLADRC over PID in overshoot reduction and disturbance rejection are reproduced on the actual prototype: overshoot decreases from 11.1% to 2.8%, and disturbance recovery time shortens from 0.9 s to 0.4 s. These are demonstrated engineering findings. We anticipate, but have not directly demonstrated, that a fast, smooth return allows the athlete to prepare immediately for the next stroke and that low overshoot avoids unpredictable ball positions that could disrupt rhythm; confirming such training benefits requires longitudinal studies with objective tennis-performance measures.

The ESO effectively estimates total disturbance (including hit impacts and friction variations) in experiments, enabling robust control without an exact model. The successful cooperative mode demonstration shows that multiple devices can be networked via Bluetooth to create rich training scenarios, laying the groundwork for future group training courses or gamified training APPs.

Beyond control performance, the signals captured by the device are relevant to training-load monitoring, the theme of this special issue. The encoder-based estimate of ball-tip speed at each hit provides a proxy of striking intensity, while the number and temporal distribution of hits per session quantify striking volume; together these constitute external-load indicators in the sense of established training-load frameworks (18, 19), analogous to hitting-load measures derived from wearable inertial sensors in tennis (20, 21). Logging these quantities across sessions would allow coaches to track load progression, positioning the device not only as a training aid but also as a monitoring tool. This link is currently conceptual, as no load data were collected during the present trial.

From an engineering-application perspective, the prototype completed a bench durability test of 5,000 consecutive hit-return cycles without mechanical or control failure, and safety is addressed through motor current limiting, an emergency-stop command in the application, and a fully enclosed housing. The bill of materials of one unit is approximately USD 450, below the price of commercial programmable tennis ball machines (typically USD 800–2,500); it should be noted, however, that ball machines feed balls whereas the present device trains striking against a physical target, so the two categories are complementary rather than directly comparable, and a systematic benchmark against commercial equipment remains future work.

The high user satisfaction scores suggest, rather than confirm, that the technical improvements translate into perceivable user benefits, an inference limited by the absence of a comparator condition and the subjective nature of the ratings. The particularly high rating for “multi-device fun” indicates that cooperative modes are a key attraction; the “response speed” score reflects perceived responsiveness and should not be conflated with the measured settling-time improvements reported in Section 3.

Limitations of this study include the manual control of hit force and direction, which introduces variability; future work will use a robotic arm to generate repeatable hits for more precise characterisation. Bluetooth communication may suffer from latency in crowded environments; upgrading to Wi-Fi or a proprietary protocol is planned. In addition, NLADRC was compared only against PID. PID was chosen as the baseline because it remains the de facto industrial standard for position servo systems and the most common controller in comparable training equipment; nevertheless, comparison with other advanced strategies (e.g., sliding mode control or linear ADRC) would further situate the proposed method and is left to future work. The user survey lacked a comparator condition (passive trainer, PID mode, or pre-trial baseline), so the reported satisfaction cannot be attributed to NLADRC-specific features rather than novelty effects; selection and expectation bias may also be present because participants were recruited from university clubs and knew they were evaluating a new device. Finally, durability, safety, and cost-effectiveness were assessed only at prototype level, and the device is intended for amateur repetitive striking practice rather than professional match-play simulation.

## 5 Conclusion

This paper presented and implemented an active return device for tennis training based on a joint motor and NLADRC. Through simulations and hardware experiments, NLADRC demonstrated superior performance over PID in low overshoot, fast response, and strong disturbance rejection, with experimental results closely matching simulations. A four-unit cooperative training system successfully demonstrated sequential and random modes, showing the feasibility of multi-target cooperative training. A preliminary survey indicates acceptable usability. The demonstrated contributions are therefore the validated device design, the superiority of NLADRC over PID for this application, and preliminary user acceptability; the device is intended for amateur repetitive striking practice, and any benefit to tennis skill or training outcomes remains to be established. Future work will optimize communication protocols, add data analysis features, and explore applications in other striking sports such as boxing and baseball.

## Data Availability

The original contributions presented in the study are included in the article/Supplementary Material, further inquiries can be directed to the corresponding author.
